# The Topological Phases of One-Dimensional Non-Hermitian Systems with Spin-Orbit Coupling of the Generalized Brillouin Zone

**DOI:** 10.3390/ma18071417

**Published:** 2025-03-23

**Authors:** Yanzhen Han, Jianxiao Liu, Shiyao Chong, Jingjing Du, Linghui Meng, Yingjie Gao

**Affiliations:** 1 College Electronics Information Engineering, Hengshui University, Hengshui 053000, China; 600457@hsnc.edu.cn (J.L.); hsxydjj@163.com (J.D.); atmeng0908@126.com (L.M.); 2 School of Electronics and Information Engineering, Jinling Institute of Technology, Nanjing 211169, China; gaoyingjie@jit.edu.cn; 3 School of Transportation, Southeast University, Nanjing 210009, China

**Keywords:** non-Hermitian, topological phases, generalized Brillouin zone

## Abstract

Revealing singular quantum phenomena in various non-Hermitian systems is a hot topic in condensed matter physics research, with the bulk-boundary correspondence being one of the core issues in non-Hermitian topological states. In addition, the spin-orbit coupling (SOC) applied to electrons moving in the electric field in the material can bring unique topological properties to the energy band of the material. We investigated the topological phase transition of a non-Hermitian Su–Schrieffer–Heeger (SSH) model with SOC in the generalized Brillouin zone (GBZ). We demonstrate that SOC can alter the position and number of phase transition points. Due to the non-Hermitian skin effect, the bulk-boundary correspondence is broken, and the local positions of zero mode and bulk eigenstates will also change. By unitary transformation, two subspaces were obtained, and the exact solution of topological phase transition was obtained in the GBZ. The exact solution of non-Hermitian systems with the Dresselhaus and Rashba types of SOC is consistent with the numerical solutions. This result can be applied to more complex non-Hermitian models, providing a strong reference for experimental researchers in topological materials.

## 1. Introduction

In the vast field of condensed matter physics, the study of topological quantum states has attracted much attention due to their unique physical properties and potential application value. In recent years, with the continuous development of topological quantum state theory, a series of new topological phases have been discovered [1,2]. Topological insulators, topological semimetals, etc., exhibit unique properties that differ from traditional materials [3,4,5,6]. These characteristics contribute to topological quantum states having broad application prospects in fields such as quantum computing and quantum communication. The topological phase of non-Hermitian systems has also become a research hotspot in recent years [7,8,9,10], especially for non-Hermitian systems with PT symmetry. The Hamiltonian of such systems does not satisfy the Hermitian condition, their eigenvalues may be complex, and the system may exhibit non-Hermitian skin effects [11,12,13,14,15,16,17,18,19,20,21], exceptional points (EPs) [22], and unique topological structures. These properties enable non-Hermitian systems to exhibit broad application potential in fields such as quantum optics and quantum information. Meanwhile, SOC, as an important mechanism connecting electron spin and orbital motion [23], has shown great potential for applications in topological materials, spintronics, and quantum computing.

The topological nontrivial characteristics of topological insulators can be caused by SOC [1,3,24,25]. In materials, the spin-orbit coupling applied to electrons moving in an electric field can be regarded as an equivalent magnetic field, which is similar to a real magnetic field and plays a key role in many spin electron materials and topological effects [23]. It may also bring unique topological properties to the material band. Under the SOC, surface/interface states without energy gaps, spin splitting, and the linear dispersion relationship may appear on the surface. These states are also protected by time reversal symmetry and are not affected by impurities and disorder. Introducing spin-orbit coupling into non-Hermitian systems, especially constructing one-dimensional models with SOC, not only deepens our understanding of non-Hermitian topological phases [26,27], but may also open up new avenues for regulating topological properties. And a non-Hermitian topological optical model based on photonic crystals has been proposed, which achieves unidirectional optical transmission of chiral edge states by regulating the gain–loss distribution [28]. The topological quantum phase transition induced by spin-orbit coupling in two-dimensional magnetic materials provides a new mechanism for regulating the quantum anomalous Hall effect [29].

We implemented a non-Hermitian SSH model incorporating SOC in cold atomic systems. Two orthogonal standing wave lasers were used to construct a one-dimensional optical lattice, trapping ultracold atoms and introducing the Rashba type SOC through Raman laser coupling technology to induce momentum-dependent spin splitting [30]. It is also possible to use spatial light modulators to adjust the depth of the optical lattice potential well in real time, construct SSH chain structures with alternating strong weak tunneling coupling [31], and achieve nonreciprocal tunneling terms through a laser-induced complex phase. Through in situ density imaging, spin-resolved time-of-flight measurements, or momentum-resolved Bragg spectroscopy, new phenomena can be observed under the synergistic effect of SOC and non-Hermitian states, such as spin-polarized skin-effect localized states, topologically protected chiral edge transport, and band singularity near exceptional points [32]. This experiment not only validates the theoretical predictions of non-Hermitian topological physics, but also lays the physical foundation for the development of new quantum devices based on cold atom platforms.

Specifically, when non-Hermitian properties are combined with one-dimensional models, not only does it enrich the theoretical framework of topological phases, but it may also enable more complex and controllable topological structures to be achieved experimentally [32,33,34,35,36,37,38,39,40,41]. In this context, expanding our research perspective to the GBZ provides a broader stage for us to delve deeper into the topological phases of non-Hermitian one-dimensional models [35]. The GBZ, as a concept unique to non-Hermitian systems, goes beyond the definition of the Brillouin zone in traditional Hermitian systems and can more accurately describe the localization characteristics of wave functions and the evolution of energy spectra in non-Hermitian systems [16,42,43,44,45]. Therefore, studying the topological phases of non-Hermitian one-dimensional models within the GBZ can not only reveal the influence of non-Hermitian properties on topological structures, but also provide a new perspective for understanding a wider range of non-Hermitian topological phenomena.

This article aims to construct a non-Hermitian one-dimensional model using the Dresselhaus and Rashba types of SOC, and we explore the influence of spin-orbit coupling on topological phase transitions, the effect of the non-Hermitian skin effect on eigenstate localization, and the topological phase in GBZ. We will use a combination of theoretical analysis and numerical simulation to deeply analyze the intrinsic relationship between non-Hermitian properties, GBZ, and topological phases. By solving the analytical and numerical solutions of the traditional Brillouin zone phase transition point, we found that all eigenstates of the open chain are located near the boundary, which is known as the ‘non-Hermitian skin effect’, which affects the local position of the eigenstates. Then, we obtained the strict solution of the topological phase transition of this model in the GBZ.

This article is organized as follows. In Section 2, we introduced the non-Hermitian model of SOC and divided the model into two subspaces through unitary transformation. In Section 3, we found that the phase transition points obtained in the traditional Brillouin zone of the system did not match the numerical calculations, so we introduced GBZ and obtained a strict solution for topological phase transition. Finally, a summary and discussion are given in Section 4.

## 2. The Non-Hermitian SSH Models

We consider a non-Hermitian SSH model with SOC, the Hamiltonian of a one-dimensional dimeric lattice in real space is

(1) $$H_{SSH}=\sum_{n,\sigma }\left(t_{1}+\gamma_{1}\right)a_{n,\sigma }^{†}b_{n,\sigma }+\left(t_{1}-\gamma_{1}\right)b_{n,\sigma }^{†}a_{n,\sigma }+\left(t_{2}-\gamma_{2}\right)a_{n+1,\sigma }^{†}b_{n,\sigma }+\left(t_{2}+\gamma_{2}\right)b_{n,\sigma }^{†}a_{n+1,\sigma },$$

where $a_{n,\sigma }^{†}$($a_{n,\sigma })$ and $b_{n,\sigma }^{†}$($b_{n,\sigma })$ are the electron creation (annihilation) operators on the sublattices A and B of the *n*-th unit cell, respectively. $\gamma_{1}$ and $\gamma_{2}$ represent the non-Hermiticity. $t_{1}$ and $t_{2}$ characterize the intracell and intercell hoppings. When the AB lattice is affected by modulated SOC, its contribution to the Hamiltonian can be described as

$$H_{SOC}=\sum_{n,\sigma }\left(\delta_{1}a_{n,\sigma }^{†}b_{n,-\sigma }-\delta_{2}a_{n+1,\sigma }^{†}b_{n,-\sigma }+h.c.\right),$$

where $\delta_{1}$ and $\delta_{2}$ denote the SOC amplitudes in the unit cell and between two adjacent unit cells, respectively. When the SOC is of the Dresselhaus type, the coupling amplitude consists of real numbers. Otherwise, when the SOC is of the Rashba type, the coupling amplitude is imaginary. The full Hamiltonian is the summation of the SSH term and the SOC term:

(2) $$H=H_{\mathrm{SSH}}+H_{\mathrm{SOC}}.$$

Using Fourier transform under periodic boundary conditions, the Hamiltonian can be easily written as $H=\sum \psi_{k}^{†}h_{k}\psi_{k}$, where $\psi_{k}^{†}={\left(a_{k,↑},a_{k,↓},b_{k,↑},b_{k,↓}\right)}^{†}$, and

(3) $$\begin{array}{c} h_{k}=\left(\begin{array}{cccc} 0 & 0 & \lambda_{1} & \mu_{1}\\ 0 & 0 & \mu_{1} & \lambda_{1}\\ \lambda_{2} & \mu_{2} & 0 & 0\\ \mu_{2} & \lambda_{2} & 0 & 0 \end{array}\right), \end{array}$$

where

$$\begin{array}{ccc} \lambda_{1} & = & \left(t_{1}+\gamma_{1}\right)+\left(t_{2}-\gamma_{2}\right)e^{-ik},\\ \lambda_{2} & = & \left(t_{1}-\gamma_{1}\right)+\left(t_{2}+\gamma_{2}\right)e^{ik},\\ \mu_{1} & = & \delta_{1}-\delta_{2}e^{-ik},\\ \mu_{2} & = & \delta_{1}-\delta_{2}e^{ik}. \end{array}$$

As a one-dimensional SOC dimerization lattice, the Hamiltonian $h_{k}$ in Equation (3) satisfies chiral symmetry $Sh_{k}S^{-1}=-h_{k}$, where $S=\tau_{3}\sigma_{0}$, and $\sigma_{0}$ is Pauli vectors. The particle-hole operator and chiral operator show the features that $S^{2}=1$.

In order to obtain the topological properties of the model and the conditions for topological phase transitions, we conducted the following analysis. We performed two unitary transformations using $U_{1}$ and $U_{2}$ [28,29]:

$$U_{1}=\frac{1}{\sqrt{2}}\left(\begin{array}{cccc} 0 & 0 & 1 & 1\\ 0 & 0 & 1 & -1\\ 1 & 1 & 0 & 0\\ 1 & -1 & 0 & 0 \end{array}\right),\;\;\;\;U_{2}=\left(\begin{array}{cccc} 0 & 1 & 0 & 0\\ 0 & 0 & 0 & 1\\ 1 & 0 & 0 & 0\\ 0 & 0 & 1 & 0 \end{array}\right),$$

then we obtain

(4) $$\begin{array}{c} \;\;\;\;U_{2}^{-1}U_{1}^{-1}h_{k}U_{1}U_{2}\\ =\left(\begin{array}{cccc} 0 & \lambda_{1}+\mu_{1} & 0 & 0\\ \lambda_{2}+\mu_{2} & 0 & 0 & 0\\ 0 & 0 & 0 & \lambda_{1}-\mu_{1}\\ 0 & 0 & \lambda_{2}-\mu_{2} & 0 \end{array}\right)\\ =h_{f}\left(k\right)⊕h_{s}\left(k\right). \end{array}$$

After unitary transformation, the non-Hermitian SSH model with SOC can be divided into two subspaces $h_{f}$ and $h_{s}$. It is worth noting that each subspace can be regarded as a regular non-Hermitian SSH model.

Next, the above model can be divided into the first subspace $h_{f}$ and the second subspace $h_{s}$ for discussion, which can be expressed as

(5) $$\begin{array}{c} h_{\alpha }=\left[\alpha_{1}+\gamma_{1}+\left(\alpha_{2}-\gamma_{2}\right)e^{-ik}\right]\sigma_{+}+\left[\alpha_{1}-\gamma_{1}+\left(\alpha_{2}+\gamma_{2}\right)e^{ik}\right]\sigma_{-}, \end{array}$$

where $\sigma_{\pm }=\frac{1}{2}\left(\sigma_{x}\pm i\sigma_{y}\right)$ and $\sigma_{x,y}$ are the Pauli matrix in the first subspace

(6) $$\begin{array}{c} \alpha_{1}=t_{1}+\delta_{1},\;\;\;\alpha_{2}=t_{2}-\delta_{2} \end{array}$$

and in the second subspace

(7) $$\begin{array}{c} \alpha_{1}=t_{1}-\delta_{1},\;\;\;\alpha_{2}=t_{2}+\delta_{2}. \end{array}$$

## 3. Topological Phase Transition

For simplicity, we can first explore the case where the SOC is of the Dresselhaus type. We diagonalize the Hamiltonian $h_{\alpha }$ in the momentum space to obtain the eigenvalues

(8) $$\begin{array}{c} E_{\alpha }\left(k\right)=\pm \sqrt{\alpha_{1}+\gamma +\left(\alpha_{2}-\gamma_{2}\right)e^{-ik}}\sqrt{\alpha_{1}-\gamma +\left(\alpha_{2}+\gamma_{2}\right)e^{ik}}. \end{array}$$

The energy gap closes at the exceptional points which requires $E_{\alpha ,\pm }=0$. Therefore, the exceptional points at the closure of the energy gap can be obtained in two subspaces: when α=f,

(9) $$\begin{array}{c} t_{1}=-t_{2}-\delta_{1}+\delta_{2}\pm \gamma_{1}\mp \gamma_{2}\;\;\;\;\left(k=0\right),\\ t_{1}=t_{2}-\delta_{1}-\delta_{2}\pm \gamma_{1}\mp \gamma_{2}\;\;\;\left(k=\pi \right), \end{array}$$

and when α=s,

(10) $$\begin{array}{c} t_{1}=-t_{2}+\delta_{1}-\delta_{2}\pm \gamma_{1}\mp \gamma_{2}\;\;\;\;\left(k=0\right),\\ t_{1}=t_{2}+\delta_{1}+\delta_{2}\pm \gamma_{1}\mp \gamma_{2}\;\;\;\;\left(k=\pi \right). \end{array}$$

**Figure 1:**
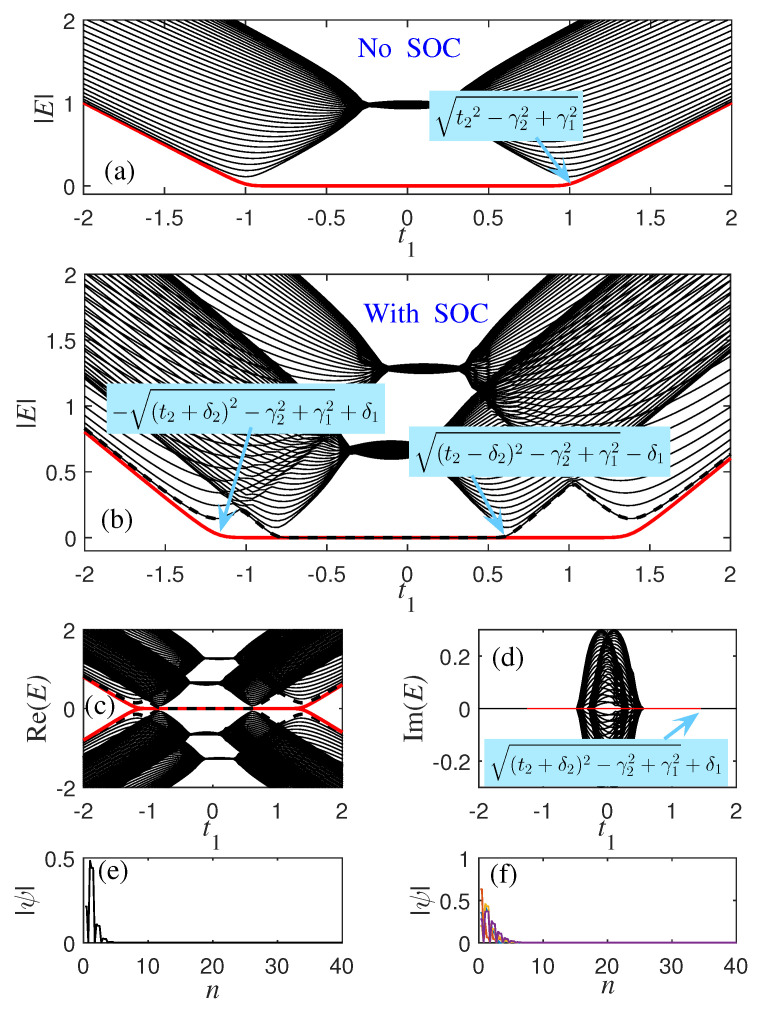
(**a**) The energy spectrum with 40 unit cells of the non-Hermitian SSH model for $H_{SSH}$ in Equation (1) under open boundary conditions. The parameters are $t_{2}=1$, $\gamma_{1}=0.3$, $\gamma_{2}=0.2$. (**b**) The energy spectrum of non-Hermitian models with SOC for $h_{k}$ in Equation (3) under open boundary conditions. The two energy gap closure points $-\sqrt{{\left(t_{2}+\delta_{2}\right)}^{2}-\gamma_{2}^{2}+\gamma_{1}^{2}}+\delta_{1}$ and $\sqrt{{\left(t_{2}-\delta_{2}\right)}^{2}-\gamma_{2}^{2}+\gamma_{1}^{2}}-\delta_{1}$. The other parameters are $\delta_{1}=0.1$, $\delta_{2}=0.3$. (**c**,**d**) The real and imaginary parts of E in (**b**). (**e**) Profile of a zero mode and (**f**) four randomly chosen bulk eigenstates with $t_{1}=0.4$ in (**b**).

In the non-Hermitian system without SOC, the positions of the energy spectrum and the phase transition point are shown in Figure 1a. According to Equations (9) and (10), when the parameters are $t_{2}=1$, $\gamma_{1}=0.3$, and $\gamma_{2}=0.2$, the phase transition point obtained by the traditional Brillouin zone should be located at $t_{1}=-1.1,-0.9,\;0.9$, and 1.1. But as shown in Figure 1a, the non-Hermitian model only has two phase transition points located near $t_{1}=\pm 1$. And when the SOC is of the Dresselhaus type, the energy spectrum of the system can be obtained by diagonalizing the Hamiltonian $h_{k}$ in Equation (3) under OBC, as shown in Figure 1b. The zero-mode line is displayed as a red solid line and a black dashed line. According to Equations (9) and (10), when the parameters are $t_{2}=1$, $\delta_{1}=0.1$, $\delta_{2}=0.3$, $\gamma_{1}=0.3$, and $\gamma_{2}=0.2$, there are eight phase transition points here, which are −1.1, −0.9, 0.9, and 1.1. $t_{1}=\pm$1.3, −1.1, −0.9, ±0.7, 0.5, and 1.5. But according to the numerical solution [Figure 1b], it can be seen that the phase transition points are about −1.2, −0.8, 0.6, and 1.4, where the energy gaps are closed ($E_{\alpha ,\pm }\neq 0$). The profile of zero mode and four randomly selected bulk eigenstates, as shown in Figure 1e,f, illustrate the non-Hermitian skin effect found in the analytical solution, where all bulk eigenstates are located near the boundary. Due to the non-Hermitian skin effect, it is not possible to obtain phase transition solutions using the usual methods.

**Figure 2:**
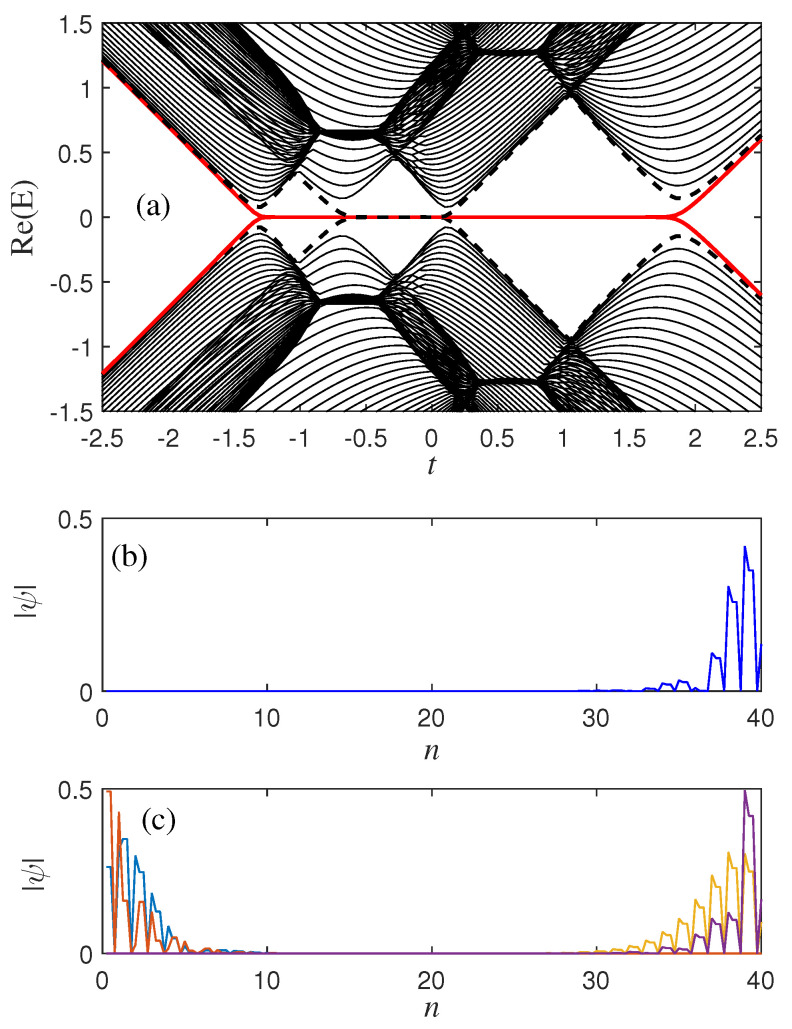
(**a**) The energy spectrum of non-Hermitian models with SOC for $\delta_{1}=0.6$. (**b**) Profile of a zero mode and (**c**) four randomly chosen bulk eigenstates. The other parameters are set as $t_{2}=1$, $\gamma_{1}=0.3$, $\gamma_{2}=0.2$, $\delta_{2}=0.3$, which are the same as the parameters in Figure 1b.

When the SOC modulation parameter $\delta_{1}=0.6$ in the unit cell, and the other parameters do not change compared with Figure 1b, we can clearly find that the phase transition point has changed, which is about −1.3, −0.6, 0.1, and 1.9 [as shown in Figure 2a]. This indicates that SOC not only changes the topological phase transition but also alters the local state of the skin effect. The non-Hermitian skin effect is closely related to topological phase transitions. When system parameters change, the presence of non-Hermitian skin effects may lead to topological phase transitions in the system. During the phase transition, the localization properties of the eigenstate undergo a sudden change, transitioning from one localized state to another.

Next, in order to obtain an analysis of the topological phase transition, we will analyze it from the GBZ. We analyze each subspace separately according to Equation (5). Assuming the length of the subsystem is *L*, the real space Schrödinger equation under open boundary conditions is

(11) $$\begin{array}{c} \left(\alpha_{2}-\gamma_{2}\right)\psi_{n-1,B}+\left(\alpha_{1}+\gamma_{1}\right)\psi_{n,B}=E_{\alpha }\psi_{n,A},\\ \left(\alpha_{1}-\gamma_{1}\right)\psi_{n,A}+\left(\alpha_{2}+\gamma_{2}\right)\psi_{n+1,A}=E_{\alpha }\psi_{n,B}. \end{array}$$

The elements of the wavefunction have the form [35]

$$\begin{array}{c} \left(\phi_{n,A},\phi_{n,B}\right)=\beta_{\alpha }^{n}\left(\phi_{A},\phi_{B}\right), \end{array}$$

which satisfy the following relationship:

(12) $$\begin{array}{c} \left[\left(\alpha_{1}+\gamma_{1}\right)+\left(\alpha_{2}-\gamma_{2}\right)\beta_{\alpha }^{-1}\right]\phi_{B}=E_{\alpha }\phi_{A},\\ \left[\left(\alpha_{1}-\gamma_{1}\right)+\left(\alpha_{2}+\gamma_{2}\right)\beta_{\alpha }\right]\phi_{A}=E_{\alpha }\phi_{B}. \end{array}$$

So, the characteristic equations of the subspace of the non-Hermitian SSH model can be obtained:

(13) $$\left[\left(\alpha_{1}+\gamma_{1}\right)+\left(\alpha_{2}-\gamma_{2}\right)\beta_{\alpha }^{-1}\right]\left[\left(\alpha_{1}-\gamma_{1}\right)+\left(\alpha_{2}+\gamma_{2}\right)\beta_{\alpha }\right]=E_{\alpha }^{2}.$$

The non-Bloch Hamiltonian of each subspace can be expressed as [45]

(14) $$\begin{array}{c} H_{\alpha }=\left(\begin{array}{cc} 0 & \left(\alpha_{1}+\gamma_{1}\right)+\left(\alpha_{2}-\gamma_{2}\right)\beta_{\alpha }^{-1}\\ \left(\alpha_{1}-\gamma_{1}\right)+\left(\alpha_{2}+\gamma_{2}\right)\beta_{\alpha } & 0 \end{array}\right). \end{array}$$

And the two solutions of Equation (13) are

(15) $$\beta_{\alpha }^{1,2}\left(E\right)=\frac{-\kappa \pm \sqrt{\kappa^{2}-4\left(\alpha_{1}^{2}-\gamma_{1}^{2}\right)\left(\alpha_{2}^{2}-\gamma_{2}^{2}\right)}}{2\left(\alpha_{1}+\gamma \right)\left(\alpha_{2}+\gamma_{2}\right)},$$

where $\kappa =\left(\alpha_{1}^{2}-\gamma_{1}^{2}\right)+\left(\alpha_{2}^{2}-\gamma_{2}^{2}\right)-E_{\alpha }^{2}$, and +(−) correspond to $\beta_{\alpha }^{1}\left(\beta_{\alpha }^{2}\right)$. When E→0,

(16) $$\beta_{\alpha ,E\to 0}^{1,2}=-\frac{\alpha_{2}-\gamma_{2}}{\alpha_{1}+\gamma_{1}},-\frac{\alpha_{1}-\gamma_{1}}{\alpha_{2}+\gamma_{2}}.$$

The trajectories of $\beta_{\alpha }^{1}$ and $\beta_{\alpha }^{2}$ satisfying $\left|\beta_{\alpha }^{1}\right|=\left|\beta_{\alpha }^{2}\right|$ constitute the GBZ. These two solutions satisfy $\frac{\left(\alpha_{2}-\gamma_{2}\right)\left(\alpha_{1}-\gamma_{1}\right)}{\left(\alpha_{1}+\gamma_{1}\right)\left(\alpha_{2}+\gamma_{2}\right)}$.

**Figure 3:**
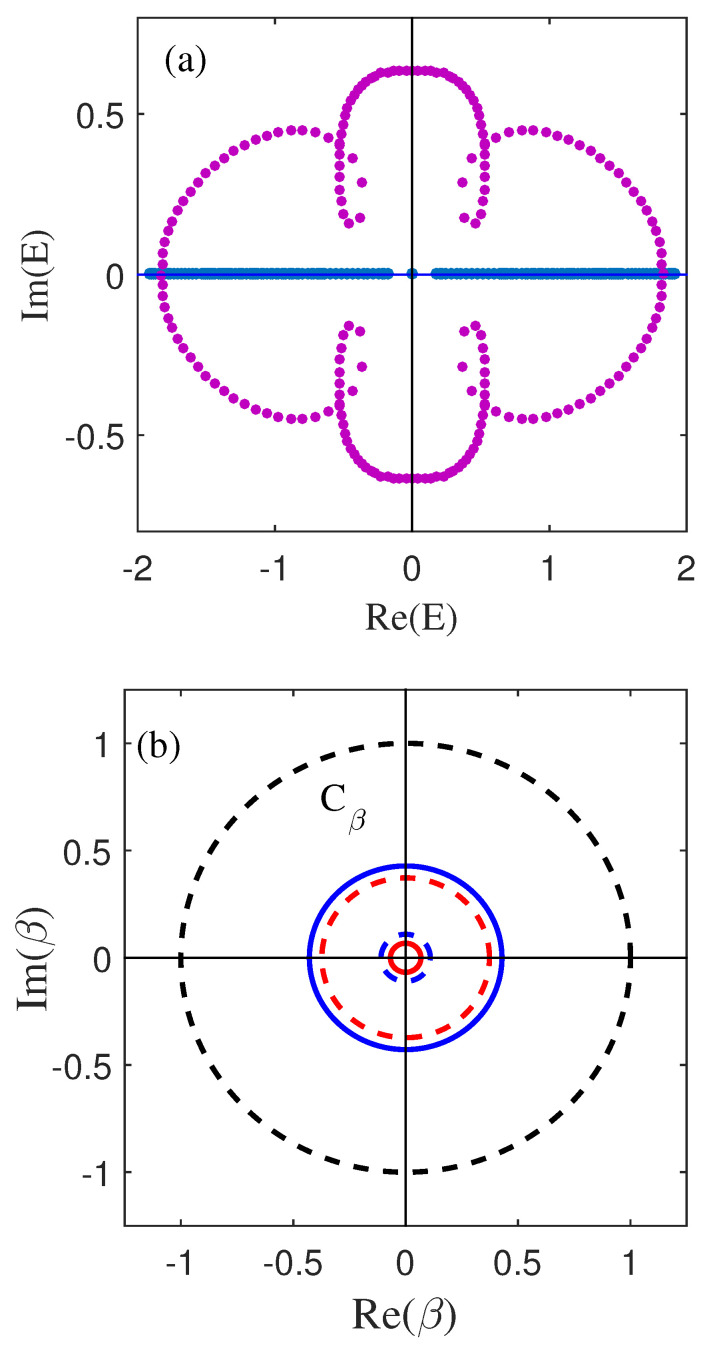
(**a**) The energy spectrum in the open boundary condition (the blue line) and in the periodic boundary condition (the red line) with $t_{1}=0.8$. (**b**) The black dotted curve represents the Brillouin zone, whereas the red lines ($\beta_{f}$) and blue lines ($\beta_{s}$) represent the GBZ. From outside to inside, $t_{1}=0.6,\;0.4,\;0.41,\;0.205$. Other parameters are the same as in Figure 1.

We can obtain

(17) $$\left|\beta_{\alpha }^{1}\right|=\left|\beta_{\alpha }^{2}\right|=r=\sqrt{\left|\frac{\left(\alpha_{2}-\gamma_{2}\right)\left(\alpha_{1}-\gamma_{1}\right)}{\left(\alpha_{1}+\gamma_{1}\right)\left(\alpha_{2}+\gamma_{2}\right)}\right|},$$

which is the GBZ of the subspace of the non-Hermitian SSH model, with a radius of *r*. As shown in Figure 3a, the energy spectrum under the open boundary condition composed of blue dots deviates from the energy spectrum under the periodic boundary condition (purple dots). The blue dots at the origin represent the two degenerate zero modes of an open chain. This deviation is due to the non-Hermitian skin effect shown in Figure 1d. As shown in Figure 3b, the complex value $\beta_{\alpha }$ of the subspace forms a closed loop $C_{\beta }$. Obviously, when $\alpha_{1}$ closes to $\gamma_{1}$, the GBZ develops a tendency to collapse into a point. In the Hermitian case, $C_{\beta }$ is the unit circle (black dashed line).

According to $\left|\beta_{\alpha }^{1}\right|=\left|\beta_{\alpha }^{2}\right|$, when E→0, we can obtain

(18) $$\left|\frac{\alpha_{2}-\gamma_{2}}{\alpha_{1}+\gamma_{1}}\right|=\left|\frac{\alpha_{1}-\gamma_{1}}{\alpha_{2}+\gamma_{2}}\right|.$$

Considering that the SOC is of the Dresselhaus type, and by inserting Equation (7) into the above equation, the topological phase transition points can be obtained from the two subspaces

(19) $$\begin{array}{c} t_{1}=\pm \sqrt{{\left(t_{2}-\delta_{2}\right)}^{2}-\gamma_{2}^{2}+\gamma_{1}^{2}}-\delta_{1},\\ t_{1}=\pm \sqrt{{\left(t_{2}+\delta_{2}\right)}^{2}-\gamma_{2}^{2}+\gamma_{1}^{2}}+\delta_{1}. \end{array}$$

When $t_{2}=1$, $\gamma_{1}=0.3$, $\gamma_{2}=0.2$, $\delta_{1}=0$, and $\delta_{2}=0$, then according to Equation (19), $t_{1}=\pm \sqrt{t_{2}^{2}-\gamma_{2}^{2}+\gamma_{1}^{2}}=\pm 1.0247$ is obtained. This is consistent with the numerical results shown in Figure 1a. We substituted the parameters from Figure 1b into Equation (19), then obtained $t_{1}=-1.2191$, $t_{1}=-0.8348$, $t_{1}=0.6348$, and t=1.4191, which are consistent with the numerical results shown in Figure 1b. This is a manifestation of the non-Bloch bulk-boundary correspondence.

**Figure 4:**
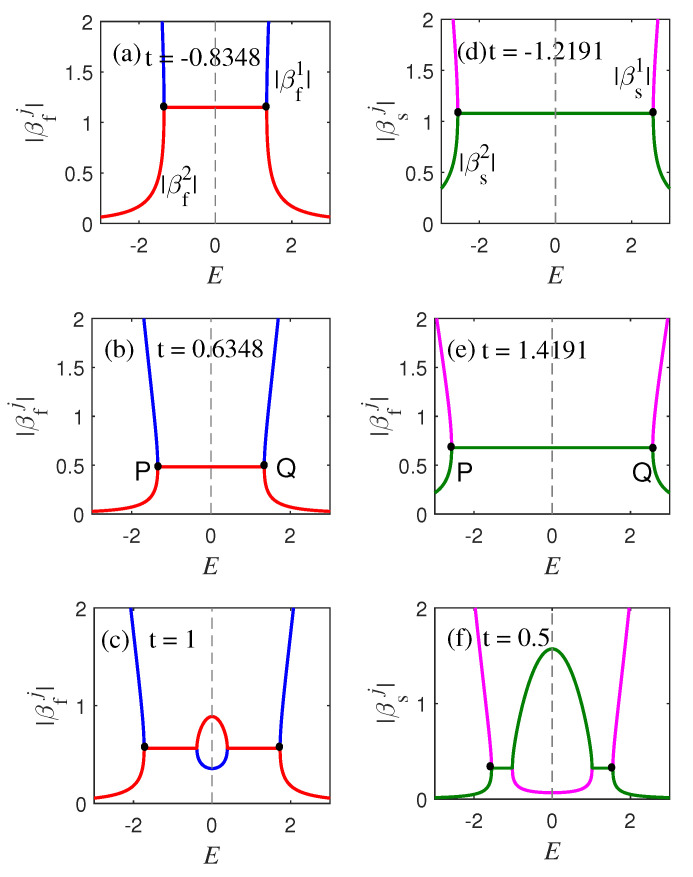
$\left|\beta_{\alpha }^{j}\right|-E$ curves from Equation (15) in two subspaces. The **left** (**right**) column diagrams correspond to the first (second) subspace, with parameters of $t_{2}=1$, $\delta_{1}=0.1$, $\delta_{2}=0.3$, $\gamma_{1}=0.3$, $\gamma_{2}=0.2$.

To rederive the transition point in Equation (15), we plotted the $\left.|\beta_{\alpha }^{j}\right.|-E$ curve in Figure 4. The left columns (a)–(c) of the figure show the variation curves of $\left.|\beta_{f}^{1}\right.|$ (blue curve) and $\left.|\beta_{f}^{2}\right.|$ (red curve) in the first subspace, whereas the right columns (d)–(f) show the variation curves of $\left.|\beta_{s}^{1}\right.|$ (magenta curve) and $\left.|\beta_{s}^{2}\right.|$ (green curve) in the second subspace. An expected relationship $\left.|\beta_{\alpha }^{1}\right.\left|=\left.|\beta_{\alpha }^{2}\right.\right|$ related to the bulk spectra was discovered on the PQ line [Figure 4a]. Whether in the first subspace or the second subspace, it can be clearly seen that the two curves are tangent in PQ, as shown in Figure 4a,b,d,e, when $t_{1}=-1.2191$, $t_{1}=-0.8348$, $t_{1}=0.6348$, and t=1.4191. This indicates that the result of Equation (19) is correct and corresponds to the result in Figure 1a.

When the SOC is of the Rashba type, which means that $\delta_{1}$ and $\delta_{2}$ are imaginary numbers, SOC can also manipulate topological phase transitions. According to Equation (18), the energy gap closure point is

$$\begin{array}{c} t_{1}=\pm \sqrt{\frac{-b+\sqrt{b^{2}-4ac}}{2a}}, \end{array}$$

where

$$\begin{array}{c} b=2\left({\left|\delta_{1}\right|}^{2}-2\gamma_{1}^{2}\right), \end{array}$$

$$\begin{array}{c} c={\left(\gamma_{1}^{2}+{\left|\delta_{1}\right|}^{2}\right)}^{2}-\left[{\left(t_{2}-\gamma_{2}\right)}^{2}+{\left|\delta_{2}\right|}^{2}\right]\left[{\left(t_{2}+\gamma_{2}\right)}^{2}+{\left|\delta_{2}\right|}^{2}\right]. \end{array}$$

**Figure 5:**
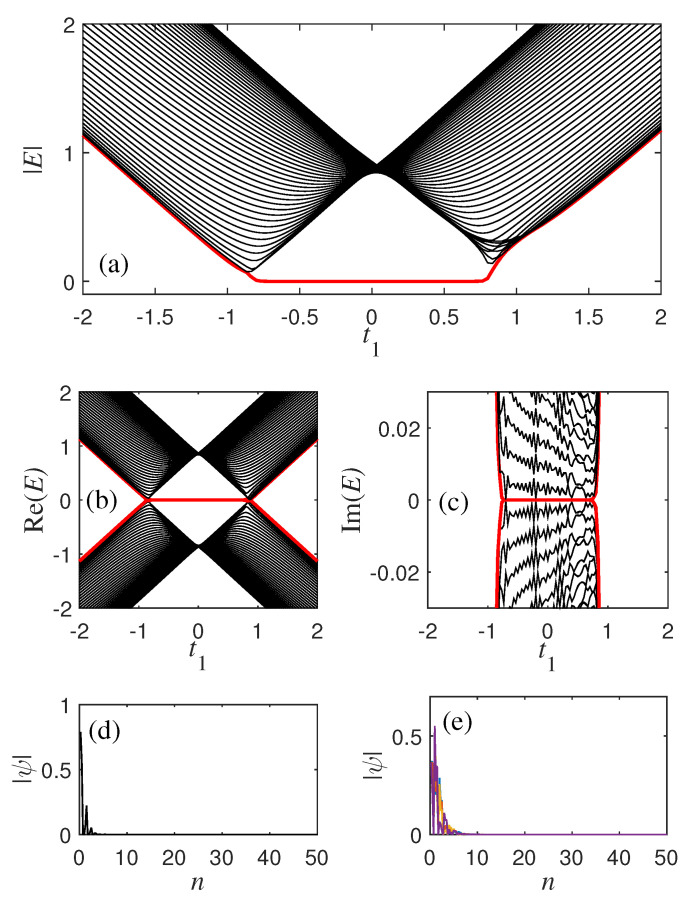
(**a**) The energy spectrum under the open boundary controlled by the Rashba SOC. (**b**,**c**) The real and imaginary parts of E with $t_{2}=1$, $\delta_{1}=0.2i$, $\delta_{2}=0.1i$, $\gamma_{1}=0.1$, $\gamma_{2}=0.5$, $t_{3}=0$. (**d**) Profile of a zero mode and (**e**) four randomly chosen bulk eigenstates with parameters $t_{1}=0.4$.

When $t_{2}=1$, $\delta_{1}=0.2i$, $\delta_{2}=0.1i$, $\gamma_{1}=0.1$, and $\gamma_{2}=0.5$, the phase transition point is t=±0.8632, which is consistent with the results in Figure 5.

## 4. Conclusions

In this work, a one-dimensional non-Hermitian model with SOC was constructed to study the physical properties corresponding to the bulk-boundary correspondence of GBZ with singular features. We studied the effect of SOC on topological phase transitions and found that SOC changes the position and number of phase transition points. When the SOC is of the Dresselhaus type, we calculated the energy spectrum of the Bloch momentum space and found that the traditional bulk-boundary correspondence was broken due to the non-Hermitian skin effect. Due to the non-Hermitian skin effect of the system, the position of the topological phase transition and the local positions of bulk eigenstates and zero mode are found to be affected after modulating SOC parameters. By introducing a closed-loop GBZ, a relationship $\left|\beta_{\alpha }^{1}\right|=\left|\beta_{\alpha }^{2}\right|$ related to the energy spectrum was obtained at the analytical solution of the topological phase transition points in two subspaces, which is consistent with the results in the Bloch momentum space. Finally, in the non-Hermitian system with the Rashba type SOC, we also obtained a strict solution for the phase transition point in GBZ. In superconducting topological materials, SOC induces topological nontrivial states by breaking spatial symmetry, such as Majorana zero modes driven by SOC in iron-based superconductors or topological superconducting surface states [46]. Non-Hermitian properties (such as dissipation or gain) can significantly alter the transport characteristics of topological superconductors, such as the robustness of topological edge states in dissipative environments [47]. These findings provide important insights for the collaborative study of non-Hermitian SSH models and SOC in cold atomic systems. Future research needs to integrate the advantages of two types of systems: using cold atom simulations to reveal the microscopic mechanisms of SOC and non-Hermitian properties (such as the spin-resolved skin effect), while drawing on the design ideas of superconducting devices (such as unidirectional topological transport), to promote the development of topological quantum computing and low-power electronics.

## Data Availability

The original contributions presented in this study are included in the article. Further inquiries can be directed to the corresponding author.
